# A Treatise on Poisons in Relation to Medical Jurisprudence, Physiology, and the Practice of Physic

**Published:** 1845-10

**Authors:** 


### ART. III.

*A Treatise on Poisons in relation to Medical Jurisprudence, Physiology, and the Practice of Physic.* By ROBERT CHRISTISON, M.D. F.R.S.E. &c. Fourth Edition.—*Edinburgh,* 1845. 8vo, pp. 986.

THIS work has evidently undergone many changes since its first appearance, but the most important change is in the enlargement of bulk and in the addition to the section on irritant poisons, especially on arsenic, of a large amount of valuable information.

On the physiological action of poisons there is but little to observe, this being a purely speculative branch of the subject. The opinions of the author appear to have become modified in respect to the action of certain poisons by sympathy. The experiments of Mr. Blake appear to have in some measure shaken the author's views respecting the action of prussic acid, by a supposed sympathetic effect on the nerves of the part with which it comes in contact. He denies, however, that Mr. Blake's experiments are sufficient to show that this substance always acts by absorption, and strongly insists upon the fact that the extreme rapidity of its operation, as observed by numerous experimentalists who had no particular theories to uphold, are adverse to this view. The poison has been observed to affect the system within a period of time, less than that which Mr. Blake has demonstrated to be necessary for the process of absorption and diffusion throughout the body. In short, the absorption of the poison cannot be denied, the occasional detection of the odour in the blood, and throughout all the cavities, is a clear proof that this process is in operation in cases of poisoning by prussic acid. But the difficulty to be overcome in solving the question, is this—the poison has been known to produce its effects in *three* seconds and even less. Again, in animals in the impregnated state while it has destroyed the life of the parent, it has not affected the young contained in the uterus. So with respect to conia, the active principle of hemlock, "this is not less prompt in its operation,—when it was injected in the form of muriate into the femoral vein of a dog, I was unable with my watch in my hand to observe an appreciable interval between the moment it was injected, and that at which the animal died,—certainly the interval did not exceed three or at the most four seconds." (p. 8.)

It seems exceedingly difficult to account for this rapid operation of poisons on the supposition that before they can affect the system, they must make the round of the circulation, an assumption which the theory of their action by absorption substantially implies; and this difficulty is increased when we observe their rapid effects, in cases in which the poison has not been directly poured into the blood. Have the observers of such cases been mistaken in their observations, and exaggerated the rapidity of operation, or do the absorption and diffusion of the poison go on with even greater rapidity than Mr. Blake's own experiments would lead us to believe? Further observations are required to determine which of these, is the correct view. In the meantime Dr. Christison leaves the question unsettled:

"It is impossible therefore to concede, that Mr. Blake's inquiries, merely because they are at variance with prior results, apparently not less precise and exact than his own, put an end to the argument which has been drawn, in favour of the existence of a sympathetic action, from the extreme swiftness of the operation of some poisons. At the same time, on a dispassionate view of the whole investigation, it must be granted to be doubtful, whether this argument can be now appealed to in its present shape with the confidence which is desirable. And on the whole, the velocity of the circulation on the one hand, and the celerity of the action of certain poisons on the other, are both of them so very great, and the comparative observation of the time occupied by the two phenomena respectively, becomes in consequence so difficult and precarious, that it seems unsafe to found upon such an inquiry a confident deduction on either side of so important a physiological question as the existence or non-existence of an action of poisons by sympathy." (pp. 10-11.)

The experiments of Liebig published since the appearance of Dr. Christison's work, may be equally cited, both by sympathists and absorptionists, in support of their views. We here quote them, as they bear immediately upon the subject now under discussion. In his lectures Liebig says, "Comparatively large quantities of hydrocyanic acid in aqueous solution, may be taken into the digestive apparatus without producing any very perceptible noxious effects, while the same quantities of the acid inhaled as vapour cause immediate death. Thus a cat for instance can bear the administration of from two to three drops of anhydrous hydrocyanic acid diluted with from four to six ounces of water, without being in the least affected by it. If you place two drops of anhydrous hydrocyanic acid in the mouth of the same cat, taking care at the same time to prevent the animal from breathing by stopping its mouth and nostrils, there is no perceptible effect produced; but the animal dies the very instant that you permit it to breathe, and consequently as soon as the vapour of the acid gets into the lungs." (Lancet, Dec. 7, 1844.)

The dilution of a poison with water is commonly considered to favour its absorption by spreading it over a large surface, and thus bringing it in contact simultaneously with the numerous absorbent mouths of the intestinal canal. We presume that Liebig has performed the experiment to which he refers by the positive manner in which he states the results; but the case seems altogether inexplicable upon the view that prussic acid acts by absorption. It would be as well before drawing any inference from a single negative result, to try the effect of administering conia, strychnia, or aconitine in an equally diluted state. Liebig evidently looks upon hydrocyanic acid as operating by its vapour only; for even the anhydrous liquid in a proportion nearly equal to one hundred grains of pharmacopœial acid, is according to his last-mentioned experiment, without any effect when placed on the tongue of a cat, provided the animal be prevented from drawing the vapour of the acid into its lungs. If this experiment be correctly reported, there is at once an end to the doctrine of operation by sympathy; for the very principle of this doctrine, is founded on the fact, that mere contact with the nervous tissue of a part, is sufficient for the production of the usual effects of the poison. The only inference which we can draw from Liebig's experiments is, that prussic acid can neither operate by absorption nor by sympathy,—it acts only by its vapour which may be so diluted with a few ounces of water as to be rendered inert. Then again, this vapour is limited in its operation: it appears as if it would only act through the lungs by becoming inspired with the air. If respiration be prevented, it is said that the anhydrous acid, so highly volatile be it remembered as to boil below a temperature of 80°, has no power of penetrating through the mucous lining of the mouth and tongue to the blood-vessels beyond,—for the cat escaped, if it did not breathe; while when the vapour passed into the lungs, it traversed the mucous membrane of the air-passages and the animal fell dead instantly. Upon this theory, if the anhydrous acid or any of its aqueous solutions were injected up the rectum, no perceptible effect "should be produced," because none of the vapour could reach the lungs, but if we mistake not the life of a person has already been destroyed by the cyanide of potassium being accidentally used as an enema, and we have not the slightest doubt that any man or animal would speedily perish, if prussic acid were employed in this manner. It is highly probable that the vapour taken into the lungs may render the operation of the poison more speedy by bringing it at once in contact with a very large mucous surface. The reputation of Liebig has given such currency to his views on all subjects, even on physiology and pathology, that his statements are often received without sufficient examination. These experiments on prussic acid,—so defective in detail—so conflicting in results, and so contrary to all those facts connected with poisoning, which have been determined by careful observers, were actually quoted in a pamphlet referring to the late case of the Queen *v.* Tawell, as going to prove that two grains of anhydrous acid, were not sufficient to destroy a human being.* Liebig may not have foreseen that such an improper inference would have been drawn; but this nevertheless only proves the danger of a man, eminent in one department of science, venturing to express opinions in another in which his information is obviously imperfect.

We cannot leave this subject without calling attention to the results very recently obtained by Dr. H. Meyer in his experiments on the action of hydrocyanic acid on animals. The acid which he used was that of Ittner,

* Remarks and Comments on the trial of John Tawell, by G. L. Strauss; London, 1845.

containing 10 per cent. of the anhydrous acid. He found—1. That the acid had a paralysing action on the peripheric nerves, i. e. it suppressed sensation and motion, and occasioned congestion with augmented secretion which was chiefly observed in the cavity of the mouth. 2. He found it to act only when received into the vascular system. On mechanically arresting the circulation, the poison did not act, although the integrity of the nervous system was preserved. On restoring the circulation, the operation of the poison was immediately observed. 3. Hydrocyanic acid does not act so rapidly as it was formerly believed. Its operation was never instantaneous. 4. Its fatal effect is owing to paralysis of the heart induced by the topical action of the blood mixed with hydrocyanic acid upon that organ. It required about thirty seconds for the poisoned blood to reach the heart and produce its paralysing effects, and it mattered not whether the poison was applied directly to the substance of the heart, or to parts remote from it. In Dr. Meyer's opinion, prussic acid may act independently of the brain or nerves, or of their intervention. It requires for its operation, absorption and diffusion until it reaches the heart. It is owing to this, in his opinion, that amphibia are less rapidly killed by this poison than mammalia,—the action of the heart in those animals being less necessary for the maintenance of life.* Nevertheless, in a certain dose the poison may act upon and paralyse the nervous system producing tetanic convulsions, congestion of the veins, and exudations in the serous cavities. It is not true, as it is generally believed, that in death from prussic acid, the blood does not coagulate. Dr. Meyer found that this liquid coagulated in the bodies of the animals which were killed in his experiments. (Schmidt's Jahrbücher, 1844.)

We have here, we believe, given the most recent facts which have been observed with respect to the operation of poisons; and we have thought it the more necessary to do this, because the work before us was completed before the publication of the results above quoted.†

Since the first appearance of Dr. Christison's work, considerable progress has been made in the chemical demonstration of numerous important poisons in the blood. Reasons were formerly advanced by toxicologists, why arsenic, mercury, and other poisons were not likely to be detected; but the improvements of chemical analysis in a few years have now rendered explanations of this kind unnecessary. It may be interesting to the practitioner to know what are the bodies which have been thus found by chemical analysis in the blood, in cases of poisoning. Dr. Christison considers that the evidence in this respect is quite satisfactory "in the instances of iodine, sal ammoniac, oxalic acid, nitre, sulphuret of

* We have often had occasion to observe that reptiles bear a strong dose of prussic acid with less apparent effect than mammalia. Snakes and frogs have remained for a long time without manifesting active symptoms of poisoning, after thirty drops of the common acid of the shops had been given to them. If the action of the heart is less material to the maintenance of life in such animals than in mammalia, it is the same with the brain and nervous system.

† The mode in which prussic acid operates, arose accidentally in the case of Tawell. It will be seen that there is still much uncertainty in respect to its operation, and further experiments are required to elucidate the subject. The modus operandi of poisons occasionally becomes a question even in the "coroner's" court. We knew an instance, lately, in which a non-medical coroner thus summed up a case of narcotic poisoning to a "highly intelligent" jury. "The fact is, gentlemen, we learn from the medical evidence, that the poison was absorbed by the *vascular functions and carried into the nerves!*"

potassium, arsenic, mercury, copper, antimony, tin, silver, zinc, bismuth, lead, hydrocyanic acid, cyanide of potassium, carbazotic acid, sulphuretted hydrogen, camphor and alcohol." (p. 19.)

In speaking of the causes which modify the action of poisons, we find the following remarks which are of some practical interest.

"In regard to the influence of chemical combination two general laws may be laid down. One is, that *poisons which only act locally, have their action much impaired or even neutralized, in their chemical combinations.* Sulphuric acid and muriatic acid on the one hand, and the two fixed alkalis on the other, possess a violent local action; but if they are united so as to form sulphates or muriates, although still very soluble, they become merely gentle laxatives. But the case is altered if either of the combining poisons also act by entering the blood. For the second general law is, that *the action of poisons which operate by entering the blood, although it may be somewhat lessened, cannot be destroyed or altered in their chemical combinations.* Morphia acts like opium if dissolved in alcohol or fixed oil; if an acid be substituted as the solvent, a salt is formed which is endowed with the same properties: the sulphate, muriate, nitrate, and acetate of morphia all act like opium. Strychnia, arsenic, hydrocyanic acid, oxalic acid, and many more come under the same denomination: Each produces its peculiar effects, with whatever substance it is combined, provided it do not become insoluble." (pp. 26-7.)

We can hardly agree to the proposition that poisons which act locally, have their action neutralized by combination,—sulphuric acid and a fixed alkali being taken as an instance. *Quoad* a corrosive effect, the action of such bodies is undoubtedly destroyed by combination, but the alkaline sulphate is not the less capable of acting as a powerful irritant in a large dose, and destroying life. Two cases are reported at p. 657, which appear to us to establish this point. The mode of action is altered by combination, but the substance does ~~not~~ become inert. We think the first general law requires some modification, since potash, which has a local action, may by chemically combining with tartaric acid, produce a salt which exerts a poisonous action, (see case p. 658;) and it would appear from what is stated at p. 227, that the author does not consider tartaric acid to be poisonous. So again the second general law,—that poisons which operate by entering the blood, may have their action somewhat lessened, but not destroyed by chemical combination, except in those cases where they become insoluble,—requires to be somewhat modified. At any rate, the following facts appear inconsistent with such a law. The author considers that arsenic chemically unites to the sesquioxide of iron to form an insoluble compound of arsenite of iron, and on this the antidotal properties of the oxide of iron in poisoning by arsenic, are presumed to depend,—so far the law appears correct; a poison liable to be absorbed, is rendered insoluble by chemical combination, and its poisonous effects are said to be suspended. Yet in the case of copper, arsenic combines with the oxide to form the highly insoluble compound known as Scheele's green, which is an active poison, properly placed by the author among arsenical poisons, as it is chiefly to arsenic that its noxious effects are due. Insolubility then does not always render the absorbable poison inert. If it be said, that the acid mucous liquids of the stomach may render it soluble, the same explanation would apply to the arsenite of iron, which, it is alleged, is rendered inert by the addition of a larger quantity of oxide of iron. This explanation, even if true with regard to arsenite of iron, can hardly be extended to arsenite of copper: for there is no reason to believe that the giving of any extra quantity of black oxide of copper, would prevent the arsenite from exerting a noxious action. The truth is, although poisons soluble in water, act with great readiness, yet a poisonous effect in a substance is certainly not necessarily dependent on its solubility in water, nor even altogether on the quantity dissolved by the acid secretions of the alimentary canal. The action of colomel, subchloride of copper and similar compounds appear, to bear out this view.

Mr. Blake has laid it down that *the salts of the same base produce the same actions, independently of the acids with which they are combined.* Dr. Christison, in adopting this view, says, and we believe correctly, that it applies not only to bases, but to acids—"such as the hydrocyanic, oxalic, arsenious, and arsenic acids." (p. 27.) In assenting to this statement, it seems to us, so far as arsenious acid is concerned, to be somewhat inconsistent with the view that oxide of iron disarms this poison of its virulence. The arsenites of iron and copper contain the same acid, and yet we are called upon to admit that the former is inert (or why recommend it as an antidote) while the latter is a virulent poison!

These facts have an important bearing on the antidotal treatment of cases of poisoning, some have supposed that it was merely necessary to neutralize a poison, such as oxalic acid, by a base; but, as in the case of arsenic, the compound may still exert a poisonous action. Carbonate of soda has been thus improperly given in poisoning by oxalic acid,—the theoretical view consisted in merely neutralizing the poison, but the real practical object is not so much to neutralize it, as to render it inert. Hence no substance can be employed as an antidote, which has itself a noxious action, although it may have the property of neutralizing and rendering insoluble the substance for which it is actually administered. We have heard of a case of poisoning by oxalic acid, in which it was seriously proposed to administer as an antidote, a solution of subacetate of lead. Treatment of this kind is founded on mere chemical theory, without reference to the physiological action of substances.* In respect to treatment, the following remarks are judicious:

"If the poison, on the other hand, besides possessing a local action, likewise acts remotely through absorption, or by an impression on the inner coat of the vessels, mere neutralization of its chemical properties is not sufficient; for we have seen above that such poisons act throughout all their chemical combinations which are soluble. Here, therefore, it is necessary that the chemical antidote render the poison insoluble or nearly so; and insoluble not only in water, but likewise in the animal fluids, more particularly the juices of the stomach. The same quality is desirable even in the antidotes for the pure corrosives; for it often happens that in their soluble combinations these substances retain some irritating, though not any corrosive power. When we try by the foregoing criterions many of the antidotes which have been proposed for various poisons, they will be found defective; and precise experiments have in recent times actually proved them to be so." (pp. 38.)

* In the Annales d'Hygiène, Juillet 1845, M. Paumet has recommended the protochloride of tin as an antidote in poisoning by corrosive sublimate. The antidote is itself an irritant, but M. Paumet states that when dissolved in fifteen parts of water, it has no irritant properties. Out of twenty-three dogs to which corrosive sublimate had been given, and subsequently this antidote, sixteen recovered and seven died. Two parts of the antidote decompose one of the poison.

Dr. Christison agrees with most toxicologists in considering that there are no constitutional antidotes as they are termed, i.e. none which operate by a counter-action. He appears, however, to look upon ammonia as being, to a certain extent, an antidote for prussic acid (p. 39); although it is very doubtful, whether it can act in any other way than as a stimulant vapour. It has utterly failed when administered internally, and in this respect follows the law on which we have above commented, i. e. prussic acid being an absorbable poison, its action is not destroyed by chemical combination. The author next adverts to Orfila's discoveries respecting the elimination of arsenic by the urine, in the cases of persons labouring under the effects of that poison. Orfila has proposed that this secretion should be artificially increased by the use of medicines, but it appears to us, that little reliance can be placed upon any mode of treatment which necessitates the absorption of the whole of the poison, as a part of it; since there can be no doubt that it is by absorption and circulation through the system that arsenic chiefly acts. An occasional examination of the urine may, however, according to Orfila's suggestion, usefully aid our prognosis.

The chapter on the evidence of general poisoning is purely medico-legal. We do not find that the author has added much to this section of his work. In speaking of the coexistence of marks of poisoning and disease in the dead body, he directs attention to some important matters which a hasty inspector or a hasty reasoner is apt to overlook. We all know by the medico-legal struggle which was lately made in favour of the culprit Tawell, what eagerness was shown by those who conducted the defence, to obtain in the cross examination of the witnesses some sort of admission to the effect that there were marks of disease in the body. The effort entirely failed; but had it succeeded, it is manifest from the numerous cases quoted by Dr. Christison (59), that certain appearances of natural disease are often found in the bodies of persons who have died from poison, without the fair medical inference of death from poison being thereby affected. In admitting the correctness of this opinion, we may observe that there is nothing for which a counsel engaged in defending a prisoner more eagerly seeks, than an admission of this kind from a medical witness. The jury are so much in his hands, and so little in the hands of a witness, (for long explanations on medical doctrines are never allowed in court) that it is easy to foresee how by dexterous management the verdict may be made to run. The only chance of the truth being brought to light in such a case (*veritas in curiâ!*) is where the symptoms and appearances produced by the disease, are widely different from those caused by the poison alleged to have been taken. On this point, the following remarks should be borne in mind, by those who are engaged in investigating a case of poisoning:

"The conclusions to be drawn from these facts are that, at all events, the medical inspector in a question of poisoning, must take care not to be hurried away by the first striking appearances of natural disease which he may observe, and so be induced to conduct the rest of the inspection superficially; and likewise, that he should not so frame his opinion on the case, as to exclude the possibility of a different cause from the apparent one, unless the appearances are such as must necessarily have been the cause of death. It may be said, that in requiring this condition for an unqualified opinion, a rigour of demonstration is exacted, which can rarely be attained in practice. But, on the one hand, it must not be forgotten, that an unqualified opinion is not always necessary; and on the other hand, although it were, I think it might be shown, if the subject did not lead to disproportionate details, that we may often approach very near the rigour of demonstration required. At present no more need be said, than that the inspector should be particularly on his guard in those cases, in which the appearances, though belonging to the effects of a deadly disease, are trifling; and still more in those in which the appearances, though great, belong to the effects of a disease, whose whole course may be latent. And I may add, that, from what I have observed of medico-legal opinions, the caution now given is strongly called for." (pp. 59-60.)

In the section on the *causes of the disappearance of poison from the body* nothing is said concerning hydrocyanic acid, although some recent trials have shown that this may become really a most important medico-legal question. The facts connected with the loss of this poison are quite as much deserving of distinct consideration, as those connected with other poisons which are here noticed.

In this section the author has omitted to consider a question of considerable importance in relation to the disappearance of poison, although it has not only been broached on numerous trials in this country, but has lately received a special examination from Orfila.* We allude to the evidence supposed to be derivable from the presence of a *certain quantity* of poison in the stomach. Dr. Christison rightly argues that the discovery of poison in the stomach is not necessary to the conviction of a person on a charge of poisoning, and adduces some cases bearing out this argument (p. 68); but he does not expose the gross fallacy implied in the assumption, that when poison is found, in order to bring about conviction for the crime, it is indispensable that the quantity detected should be *sufficient to destroy life.* Any proposition more preposterous than this, it is impossible to conceive. Our author shows that in undoubted cases of poisoning, either the poison may not be detected at all, or only in minute fractional proportions (p. 62). The cause of its disappearance, may have been vomiting and purging, absorption or decomposition. Facts of this description, known only to medical men, are sometimes kept in the back-ground in a Court of law, where a certain purpose is to be served; and a counsel is allowed to use his privilege in defence, so far as not merely to conceal them, but to produce a false impression in the minds of the jury. Thus we find the common argument employed—there was not sufficient poison found in the stomach to occasion death—therefore (!) the deceased could not have died from poison! This was an essential part of the defence in the case of Tawell. Admitting, as we do, the full privilege of a counsel to use and misuse as he pleases *legal* doctrines, it is entirely another question, when he resorts to a complete perversion of *medical* facts—involving not only a suppression of the truth, but a suggestion of what is so manifestly erroneous, as scarcely to require a serious argument for its refutation. Orfila quotes no less than eight criminal trials in which this was allowed to become a debated question in France; and it is almost invariably the practice in England, to ask a medical witness whether he found *sufficient* poison in the stomach *to cause death.* If he answers in the negative, there is generally an eloquent appeal for an acquittal to a jury, who are not informed that the poison found in the stomach is only the *surplus* of that which has really caused death! For reasons already assigned, poison may not be found after death, or in

* Annales d'Hygiène, tome 33, p. 347; Avril 1845.

very minute quantity, or the poison may be of a nature to render it impossible to determine the quantity present, as in the case of strychnia. There are many poisons, which have probably not yet been taken in their smallest fatal doses, e. g. arsenic and corrosive sublimate. Does it therefore follow that, if the quantity found should be less than can be shown to have already killed, that the accused is to be acquitted? Common sense would suggest that such a rule could never be carried into operation. But let us take one of Orfila's illustrations: Oxalic acid has not yet destroyed life in a smaller dose than half an ounce, and it is very doubtful whether two drachms would kill. Is it therefore to be admitted, that in a case of homicidal poisoning by oxalic acid, the medical witness must always produce two drachms at least of crystallized oxalic acid from the contents of the stomach, or that the question of poisoning cannot be entertained? Either this inference must be true, or the greater part of the defence in Tawell's case, to take that as a type of the class, was founded on a simple absurdity which the veriest tyro could expose.

The remarks upon the evidence derivable from experiments on animals, are highly judicious. We agree with the author that these experiments are very equivocal; and that no skilful toxicologist will put himself in the way of delivering an opinion on the force of such evidence. In some recent medico-legal trials, evidence from this source has been carried to a most absurd and unreasonable extent. In the case of Belany the medical witnesses declared that poisoning by prussic acid was accompanied by a shriek as the last act of life, and that this was the immediate precursor of insensibility, (see No. XXXVI, p. 560.) It is obvious that a most material question may be raised on a point of this nature. The absence of the shriek may be taken to negative the fact of poisoning; and all sorts of errors committed, leading to the conviction or acquittal of prisoners on false grounds. In Tawell's case the poetical name of "*death-shriek*" was given by the counsel, to this supposed invariable accompaniment of poisoning by prussic acid. It is almost needless to say that there is no foundation whatever for adopting this theory. The most experienced toxicologists, such as our author, with Orfila and others, would not have failed to record such an extraordinary, and as it was assumed well-marked character of poisoning. But in truth the statement scarcely requires a serious refutation. The witnesses who spoke of this shriek or cry based their evidence upon some experiments on horses and dogs; and because a cry of pain or a shriek was uttered by the animals, the hasty inference was drawn that the same effect was produced in the human subject. The error had currency for a time, and as we perceive, deceived the counsel engaged at Tawell's trial. Such errors are of course only liable to be corrected by accident; but the correction may be regarded in this case as complete; for four instances of poisoning by prussic acid in the human subject have occurred in the presence or hearing of others without any shriek being made—in one case the individual was heard to fall without any cry, in the others there was only a gasping sound heard.* Independently of these facts, it is quite certain that animals often die from the effects of prussic acid without making any shriek or cry. Another improper use of such experiments has been, among some witnesses, the inference that because animals generally suffer the most violent tetanic spasms when under the

* Medical Gazette, vol. xxxv, pp. 859, 895, and vol. xxxvi, p. 462; Provincial Medical and Surgical Journal, Sept. 1844, p. 398; ib. July 23, 1845, p. 461.

influence of prussic acid, the same effect would be produced in the human subject; ergo, in death from this poison, the body of the deceased should always be found in a convulsed attitude or it must have been interfered with. Such an inference is in opposition to numerous observations, but in the mean time, it shows us to what hazardous assertions such experiments may lead.

In treating of the classification of poisons we are glad to perceive that the author has published the interesting statistical tables on poisoning, which were a few years since drawn up by the Governments of England and France. (p. 108.) Above one third of all the cases of poisoning in England are caused by arsenic, and a like number by opium and its preparations. It is to be regretted that similar tables are not issued yearly, although we must admit that they convey rather an imperfect idea of the extent to which the crime of poisoning prevails in this country: 1, because they only comprise the deaths from poison generally; and 2, because with respect to the individual poisons, they are manifestly defective. Some recent disclosures have shown that under the present system of conducting coroner's inquests, i. e. without post-mortem examinations, which are rarely performed unless the rumour of poisoning be so strong, that it would become a public disgrace to neglect it;—persons die from poison, they are buried, and their deaths are registered as from natural disease. Two such cases have come within our knowledge during the present year, and their occurrence argues a very defective state of medical police in this country.*

In the pamphlet on Borough Inquests by Dr. Birt Davies, we find some remarks so apposite to the subject which we are here discussing, that we think it proper to quote them:

"The three following cases which have occurred within this borough, strongly manifest the necessity for more extensive post-mortem examinations.

"1. A person was found either dead or dying, more than one medical man of eminence seeing the body, within a few minutes of the seizure, pronounced the party to have fallen a victim to apoplexy. It was subsequently proved that the death arose from taking hydrocyanic acid.

"2. A person died in what was considered by the surgeon and physician attending, to have been a fit. Opium was found in the stomach.

"3. A person was attended by a physician and surgeon for some hours,—the illness and death were ascribed to and treated by them for apoplexy; but it was proved beyond all doubt that he died from laudanum."

Such cases as these strongly establish the necessity for not suffering a small consideration of expense to deprive the act of suicide or murder of a most salutary check, i. e. the certainty of discovery. In one of the instances to which we have above referred, the murder of the second child by poison, would assuredly have been prevented had a post-mortem examination been made at the inquest held on the body of the first. The latter, although killed by poison, as it was afterwards proved, was pronounced by the verdict to have died "a natural death," and the registration made out accordingly! So much for the security of a coroner's inquest!

Dr. Christison gives a very complete account of those morbid conditions of the body, which either by the symptoms or post-mortem appearances attending them, closely simulate the effects of irritant poisons. Upon these

* See also a case of poisoning, in which the coroner refused to allow an inspection although it was imperatively demanded. Provincial Medical and Surgical Journal, July 9, 1845, p. 445.

it is unnecessary to make any remark, especially as there is but little which is new in this portion of the work.

In treating of the chemical processes for detecting sulphuric acid in complex organic mixtures, he observes, that although simple at first sight, it is one of the most difficult problems in medico-legal chemistry. Most of the difficulties, however, are really of an artificial kind, they are not very likely to be encountered in practice; and the only object in drawing the attention of medical men to them, is to put them on their guard on a criminal trial for poisoning by sulphuric acid, against the attempts which the *medical* counsel for the defence may make to embarrass their testimony. The mouth, gullet, and stomach may strongly indicate the action of the acid, but when the witness speaks of having detected the poison in the stomach, he may be met with the question, whether Epsom salts (which the deceased may have taken before death) mixed with lemon juice or vinegar (which he may not have taken at all) would not give the same results with the tests as sulphuric acid itself—a question which he is assuredly bound to answer in the affirmative. Thus, then, one of the first steps in an analysis of this kind, is to determine either that there is no neutral sulphate present in the stomach, or that it exists only in small quantity. In poisoning by sulphuric acid, it is scarcely possible for a case to rest exclusively on chemical evidence, or the witness might find some difficulty in explaining to the satisfaction of a jury, these chemical subtleties. On the absorption of the mineral acids we find the following:

"Orfila has proved that sulphuric acid, as well as the two other mineral acids, is absorbed; for they may be detected in the urine, when they are introduced either into the stomach or through a wound. He could not succeed, however, in detecting any of them in the liver or spleen; in which organs it will be seen hereafter, that various other poisons may be discovered by chemical analysis. But Mr. Scoffren seems to have found sulphuric acid in the kidney, even although the individual survived the taking of the poison nearly two days. It is also worthy of remark, that, as will be proved presently, these acids may pass through the coats of the stomach by transudation, and so be found on the surface of the other organs in the belly." (pp. 159-60.)

In speaking of the tests for nitric acid we find the following: "The most convenient process consists in first ascertaining the acidity of the fluid, then neutralizing it with potash, and heating the residue in a tube with sulphuric acid. The vapour disengaged, if abundant, may be known by its orange colour in the tube and its odour." (p. 178.) On this we have to remark, that unless the nitre be in some quantity, sulphuric acid will not act in the manner stated. If the saline residue be drenched with the acid there may be little or no red vapour produced: the addition of a small quantity of metallic copper, would however readily cause its evolution under all circumstances, even without the aid of a spirit-lamp.

It is well known that great differences of opinion exist among medical men respecting the toxicological effects of iodide of potassium. Some look upon it as an active poison and proscribe it, others regard it as a safe medicine and use it unsparingly. It may be as well to state what a good authority has been able to collect in relation to this disputed question:

"Discrepant accounts have been given of the effects of iodide of potassium on man. When first introduced into medicine, it was conceived to be an active poison, not much inferior to iodine itself. Many, however, have since had an opportunity of observing that it is in general by no means so energetic. Its medicinal doses were gradually raised from one grain to five, ten, twenty grains; and at last Dr. Elliotson gave to not a few patients so much as two, four, or even six drachms daily in divided doses, without observing any remarkable effect. These and other similar observations however were made at a period when the salt used in British practice was much adulterated, often indeed containing eighty or ninety per cent. of impurity; at the same time it does appear that large doses of a pure salt have been occasionally taken with impunity. On the other hand it has evidently in some instances acted with great force. Mr. Alfred Taylor mentions a case, on the authority of Mr. Erichsen, where five grains produced alarming dyspnœa, attended with inflammation of the nostrils and conjunctiva of the eyes. An instance has been published where twelve grains in four doses occasioned shivering, vomiting, purging, general fever, and extreme prostration; and the purging continued for some days. Dr. Moore Neligan informs me he met with the case of an elderly lady in 1841, who, on taking three five-grain doses for two days, while labouring under irregular gout, was seized with severe headache, thirst, and swelling of the face; which symptoms were succeeded in two days by swelling of the tongue, ulceration of the gums, and profuse salivation for a week. Dr. Lawrie says he has known two grains and a half given thrice in one day, followed by great dyspnœa and irritation in the throat; and is even inclined to think that death resulted on two occasions from repeated medicinal doses. It would farther appear from some important researches made in France, that the protracted use of iodide of potassium in small doses with the food, may produce serious derangement of the health,—swelling of the face, headache, urgent thirst, inflammation of the throat, violent colic pains, and frequently bloody diarrhea. A disease characterized by the symptoms now described appeared repeatedly as an epidemic a few years ago in various parts of France, and spread so widely in one parish, that not less than a sixth of the whole population were attacked. After several careful investigations, it seems to have been fully proved that the affection was owing to the use of salt fraudulently adulterated with an impure salt, obtained from kelp after the separation of carbonate of soda, and consequently impregnated with an appreciable proportion of hydriodate of potass.

"It is difficult to arrive at any satisfactory conclusion from these statements as to the nature and energy of the action of this salt as a poison. But on the whole it appears to be not in general very active; and the few instances of unusual activity which have occurred may probably be put to the account of idiosyncrasy." (pp. 200-2.)

In speaking of the vegetable acids, Dr. Christison observes, that tartaric and citric acids may be taken in considerable quantities without injury. He quotes an instance (p. 227) where a person took six drachms of tartaric acid (without the carbonate of potash which should have accompanied it), in twenty-four hours without inconvenience. This may well have been, as from the statement, the acid was obviously taken in divided doses. A case has, however, recently occurred in London which was the subject of a trial for manslaughter, where one ounce of the acid taken by mistake for the compound tartrate of potash and soda, destroyed the life of an adult. Alarming symptoms appeared immediately after the deceased had swallowed the dose, but death did not take place until nine days afterwards. This case then proves that tartaric acid is an irritant poison, when taken in a sufficiently large dose, although by no means so energetic as oxalic acid.

The section on arsenic is undoubtedly the most important in the work. It occupies one hundred and twenty pages, and may be considered as comprising a complete history of that poison. As a reducing agent for arsenious acid, the author now recommends a mixture of crystals of carbonate of soda powdered and incinerated with one eighth of charcoal.

There is a better way, it appears to us, of obtaining this soda-flux, namely, by neutralizing tartaric acid with a solution of carbonate of soda evaporating to dryness, and incinerating in a closed platina crucible. An excellent reducing agent is thereby obtained, in which the charcoal is more finely divided, and more perfectly intermixed with the alkaline carbonate, than it can possibly be by the artificial process above recommended. There is a sort of fashion about the selection of reducing substances for arsenic, and latterly the use of cyanide of potassium has been strongly recommended. By means of it a very good metallic crust is obtained; but the salt has all the disadvantages of the black flux in being deliquescent. Dr. Christison prefers the reduction test to many of the new processes which have been introduced for the detection of arsenic. There is no doubt of the exceeding delicacy and certainty of this test. Any quantity of arsenious acid visible to the eye, may be brought to the metallic state by a little dexterous manipulation. In such small quantities, however, charcoal only should be employed as the reducing agent.

Dr. Christison objects to the term test, as applied to the *processes* for separating metallic arsenic, discovered by Marsh and Reinsch. In strictness of language, he is probably correct, but the use of the word test, is universal and is not liable to give rise to any serious mistake, as all know what is intended by it under the circumstances. The following remarks on the objections which have been urged to the employment of Marsh's test are of practical value:

"The discovery of Mr. Marsh had not been long made before the test in its original simple form was found liable to divers important fallacies. It appeared for example, that antimony yields very nearly the same appearance of metallic crust and of white powder, according to the position of the porcelain in the flame; that some porcelains glazed with oxide of zinc are similarly stained by a flame of simple hydrogen gas; that a great variety of metallic salts, if spirted up into the exit-tube, undergo reduction in the flame, and cause imitative stains on the porcelain; that iron salts seem to form stains from the same chemical action as what occurs in the case of arsenic; and that certain compounds of phosphorous acid with ammonia and animal matter, or even mere animal matters themselves, will in some circumstances produce a stain more or less similar to that which is occasioned by arsenic.

"There is no doubt, that the resemblance of most of these spurious stains to an arsenical crust has been much exaggerated. But still the similarity is sufficient to satisfy every impartial judge, that the mere production of a brilliant metallic, or white powdery stain, or both, upon porcelain is not conclusive evidence of the detection of arsenic in medico-legal inquiries. It is strong presumptive evidence; and the non-production of such stains is absolute proof that arsenic is not present. But in order to obtain irrefragable proof of its presence, the substance which forms the crusts and stains must be subjected to farther examination. And such is the object of the supplementary methods in the process detailed above. That process is perfectly free of fallacy: no substance yet known but arsenic, can yield the succession of phenomena which have been detailed. My opinion farther is, that the process may be safely simplified by withdrawing Berzelius' supplementary test of reduction in the exit-tube, and retaining the test of oxidation only, with the examination of the oxide by liquid reagents. I have retained the former in deference to the opinion expressed by a committee appointed by the French Institute, who examined the whole subject with unwearied zeal, but who, it may be observed, seem never to have had in their view the check-test of oxidation; which, with the consecutive tests, is superior in conclusiveness to the check of reduction only." (pp. 270-1.)

With respect to Reinsch's process we do not quite agree with the author's statement that the liquid containing the hydrochloric acid must be heated to near ebullition before the copper is introduced—"otherwise the copper becomes tarnished though arsenic be not present." (p. 272.) This tarnishing of the metal according to our observation, is only likely to occur where the acid is impure or too much of it is employed. We have seen tarnished pieces of copper rendered bright by boiling them in the diluted acid, and thus made more fit for the experiment. Many experimentalists have failed in the employment of this test, owing to their not having waited a sufficient time for the production of an arsenical deposit. "In the feeblest solutions, ten or fifteen minutes elapse before arsenic is visibly deposited, and forty minutes should be allowed for complete deposition; but in strong solutions, the action takes place in a few seconds." (p. 272.) Long boiling in hydrochloric acid is apt to detach the film, and the cutting of the copper into slips after the deposit, as recommended by the author, is also apt to cause a loss of arsenic. It has been lately proposed not to heat the copper with the film of arsenic upon it, in order to procure octohedral crystals of arsenious acid, but to scrape off the metal, collect it as a powder, and gently heat it in a tube. A larger quantity of arsenic may in this way be accumulated for sublimation. Dr. Christison has obtained satisfactory evidence of the presence of arsenic in the organic tissues, after several months' interment, even when in very small proportion, by the process of Reinsch. He has not found it necessary to incinerate for this purpose. His plan has been to cut the soft solids into small fragments, add distilled water if necessary, then hydrochloric acid to the amount of one tenth of the whole mixture,—and more if the subject of analysis were decayed and ammoniacal, so that there may always be an excess of acid. The mixture is then boiled for an hour, until all soft solids be either dissolved or broken down into fine flakes and grains. Filter through calico, bring the filtered fluid again to the boiling point, and then add the copper. We quote this description because medical practitioners are now expected to be competent to undertake the analysis of the tissues of the body for arsenic, and it is much more simple than the French processes by incineration. We agree with the author, that if the latter be adopted, the carbonization of the organic matter by sulphuric acid is decidedly preferable to Orfila's plan of incinerating with nitre or nitric acid. The latter plan answers very well with Marsh's, but not with Reinsch's process. It should be borne in mind, that in examining for arsenic in the tissues, the organs which are most likely to yield it in the largest quantity, are the liver and the spleen.

No case of poisoning by arsenic now comes to trial in which the most ingenious objections, founded on Orfila's researches and opinions, are not urged to the chemical evidence of the presence of the poison. Rightly or wrongly, applicable or inapplicable, they are invariably raised by a counsel in defence, as so many *chevaux de bataille,* for the purpose not of confuting but of embarrassing chemical evidence. A momentary triumph on a point of this nature may have a wonderful effect with a jury, even although the triumph may be founded in error. It may be beyond their power to follow the intellectual conflict; but they will proceed to draw the rough conclusion that there must have been something wrong, or the medical witness would not have been embarrassed by the questions. Medical men, conscious of the correctness of their views, are under such circumstances apt to become provoked at the sophistry opposed to them: but there is no remedy for this,—they should remember that the grand object of a counsel in defending a prisoner, is not the development of truth or the vindication of any abstract principle of justice, but the *acquittal* of the party for whom he appears!

In reference to the objection that arsenic may exist in the apparatus employed for the detection of the poison, as for example, in cast-iron where this is used for an evaporating vessel, the author properly censures the proceedings of the French chemists in the celebrated procès Laffarge.

"Besides, a false importance has been attached to the enthusiastic analyses of the whole human carcase, with which some French chemists have been astounding the minds of the scientific world, as well as the vulgar, on the occasion of certain late trials for poisoning. I confess I could not find fault with a jury, who might decline to put faith in the evidence of poisoning with arsenic, when the analyst, after boiling an entire body, with many gallons of water, in a huge iron cauldron, making use of whole pounds of sulphuric acid, nitric acid, and nitre, and toiling for days and weeks at the process, could do no more than produce minute traces of the poison. What man of common sense will believe that, with such bulky materials and crude apparatus, it is possible to guard to a certainty against the accidental admission of a little arsenic? At all events I am much mistaken, if any British jury would condemn a prisoner on such evidence,—or any British chemist find fault with them for declining to do so." (pp. 280-1.)

The sum of our information respecting the existence of arsenic as a natural constituent of the human body, is comprised in the following passage:

"This startling proposition was first advanced by M. Couerbe, and by Professor Orfila soon afterwards. The latter subsequently stated that it exists only in the bones, and not in any of the soft solids. It is now clear however that both of these experimentalists must have committed an error. Orfila himself admits that his early researches are vitiated by the subsequent discovery of arsenic in some kinds of sulphuric acid; and all recent attempts by others to obtain his results have failed. Thus MM. Flandin and Danger could not detect arsenic in any part of the human body, when it had not been administered; Pfaff was unable to detect an atom of it in the bones of man or the lower animals by Orfila's own process: Dr. Rees was equally unsuccessful. And in 1841 a Committee of the French Institute, who superintended the performance of an analysis in three cases by Orfila, reported that he failed in every instance to find a trace of arsenic, by a process which could detect a 65th part of a grain intentionally mixed with an avoirdupois pound of bones.

"There is the strongest possible presumption therefore that human bones never contain any arsenic. And besides, supposing they did, the source of fallacy would be utterly insignificant; for, when it becomes necessary to search for arsenic absorbed into the textures of the body, it is never necessary to have recourse to the bones." (pp. 281-2.)

In treating of the symptoms and appearances produced by arsenic, some interesting cases are added, but we find nothing requiring particular notice.

In speaking of the *treatment* of cases of poisoning with arsenic, Dr. Christison regards the hydrated oxide of iron as a chemical and not as a mechanical antidote, the opinion which he formerly entertained. "In confirmation of these views, and as a fact worthy of investigation on its own account, it is worthy of notice that according to Dr. Duflos, the acetate of sesquioxide of iron answers equally well as an antidote with the sesquioxide itself. It precipitates both arsenious and arsenic acids from every state of solution, and always the more quickly the more the solution is diluted; and the coexistence of acetic acid is no obstacle to this action taking place. More recently Professor Orfila has called in question the absolute efficacy, generally ascribed to the sesquioxide of iron. He alleges that the arsenical compound formed, although insoluble in water, is soluble to some extent in the gastric juices, and is consequently a poison to animals, that the sesquioxide is, therefore, only partial in its operation as a remedy; but yet that the influence of the animal fluids in the stomach, may be overcome by giving it in excess, so that as fast as the compound is dissolved, it is thrown down again." (pp. 365.)

Without admitting the correctness of Orfila's chemical explanation, we think he is right in saying, that the sesquioxide has only a very partial operation as a remedy. We must object to this ready acceptance of Dr. Duflos' statement, concerning the efficacy of acetate of iron, without a single experiment in support of it. Acetate of iron, either concentrated or diluted, precipitates arsenic acid and the arseniates in a white gelatinous mass, it also partially precipitates the arsenites, according to their degree of alkalinity: but according to our observations, it has not the least effect upon arsenious acid, whether in solution or in powder,—whether the acetate be concentrated or diluted, or whether it be pure acetate of the sesquioxide or of the mixed oxides. We should have preferred seeing a statement of Dr. Christison's own results on this subject, to the quotation of those of Dr. Duflos, which are hereby made to acquire greater authority than we believe would otherwise be conceded to them.

Among the tests for mercury we do not find that the author makes any mention of the ingenious process lately devised by Dr. Frampton for separating mercury by metallic silver. The section on mercurial salivation, and on the diagnosis between this state and cancrum oris is full of practical matter, and deserves the serious consideration of those who are called upon to give evidence in cases of suspected mercurial poisoning. In the chapter on lead, we find the addition of the author's late interesting researches on the action of air and water on that metal. To the chapter on mechanical irritants he has appended some remarks on certain saline substances, which have been observed to exert a poisonous action in large doses. This addition was rendered the more necessary by the fact, that questions had arisen at one or two recent trials respecting the operation of such substances.

In respect to the effects of opium on young children, Dr. Christison observes:

"My colleague, Dr. Alison, tells me he has met with a case where an infant a few weeks old died with all the symptoms of poisoning with opium after receiving four drops of laudanum, and that he has repeatedly seen unpleasantly deep sleep induced by only two drops.—These remarks being kept in view, it will, I suspect, be difficult to go along with an opinion against poisoning expressed by a German medico-legal physician in the following circumstances. A child's maid, pursuant to a common but dangerous custom among nurses, gave a healthy infant, four weeks old, an anodyne draught to quiet its screams. The infant soon fell fast asleep, but died comatose in twelve hours. There was not any appearance of note in the dead body; and the child was therefore universally thought to have been killed by the draught. But the inspecting physician declared this to be impossible, as the draught contained only an eighth of a grain of opium and as much hyoscyamus. In the first edition of this work an opinion was expressed to the same purport. But the facts stated above throw doubt on its accuracy, and rather show that the dose was sufficient in the circumstances to occasion death." (p. 714.)

So many inquests are held on the bodies of young children accidentally poisoned by opium, that the above extract is worthy of attentive perusal. There is too general a disposition to screen the party making the mistake, on the ground that the dose of opiate given was too small to produce serious consequences. Some space is devoted to the effects of opium eating on health, and its bearings on life insurance. No less than twenty-five cases are reported, the results of which tend to show in spite of a prejudice prevailing to the contrary, "that the practice of opium eating is not so injurious to health, and an opium eater's life, not so uninsurable as is commonly thought; and that an insured person, who did not make known this habit, could scarcely be considered guilty of concealment to the effect of voiding his insurance." (p. 721.) The case of Lord Mar, it seems, came to no satisfactory decision. The habit of opium-eating had not been made known to the insurance company at the time of the insurance, and they resisted payment on the ground of material concealment. They lost their cause, however, owing to some technical point, and on the motion for a new trial, a compromise was afterwards entered into.

Much has been lately said and written on poisoning by hydrocyanic acid. The trials of Belany and Tawell, more especially the latter, have caused all English works on Toxicology to undergo a strict examination. Some objections were taken to certain statements contained in them, but we believe chiefly by those who were unable to find in them, any facts in support of the absurd defence adopted in Tawell's case. On the contrary, the facts and opinions were such as to make the guilt of an obviously guilty man, still more clear. While, however, principles could not be attacked, supposed errors of omission and commission were denounced; at the same time there was an astonishing blindness to the errors and defects of the French writers, upon whom the counsel for the defence appears to have relied.

The two main circumstances which have been objected to as erroneous, related to the evidence from the *odour* of the poison, and the *smallest dose* which was required to destroy life. Dr. Christison says, "the peculiar odour of the acid is a very characteristic and delicate test of its presence. According to Orfila, the smell is perceptible when no chemical reagent is delicate enough to detect it. But I doubt the accuracy of this statement; and may further observe that I have known some persons *nearly insensible of any smell,* even in a specimen which was tolerably strong. Hence when the odour is resorted to as a test, it ought to be tried by several persons." (p. 753.) There is no doubt that many persons are insusceptible of the odour of prussic acid; and this may be one explanation of the very discrepant statements which have been published on this subject. Dr. Christison asserts that it was not perceptible in the blood or any other part of the body of the Parisian epileptics, (p. 773,) a statement which has been condemned as erroneous on Orfila's authority, but which is fully borne out by the report given by Orfila himself in the 'Annales d'Hygiène,' for 1829. The main point is this: the eminent physicians who inspected the body shortly after death, Adelon, Marc, and Marjolin, could perceive no smell of prussic acid; but in a report published fourteen years afterwards, Orfila states that Gay Lussac and himself detected the odour not at the time of the inspection, but *eight* days after death. We agree with Dr. Christison in placing more confidence in the original report; and in thinking that if two persons could detect prussic acid by the smell, in the contents of a stomach *eight days* after death, it is impossible to understand how at least if the odour were present one out of three experienced physicians should not have discovered it at the time of making the inspection! We are inclined to think that Gay Lussac and Orfila were more likely to have been deceived respecting the odour, than that either Adelon, Marc, or Marjolin should have entirely failed to detect the least trace of it. Had prussic acid been obtained by distillation from the contents of the stomach, a different conclusion might have been drawn, but we do not find this to be any where stated.

The absurdity of the defence of Tawell lay in this: that knowing the great volatility and diffusibility of the poison, also that it is liable to be absorbed and may destroy life in a comparatively small dose,—there is nothing medically speaking impossible, or even extraordinary that a person should now and then be poisoned by a small dose of prussic acid without any odour being perceptible in the stomach. While in the abstract, a principle of this kind could not be objected to, an attempt was made to mislead the Court by pointing out in the report of a particular case, some discrepancies among English and French writers. If the cases of the Parisian epileptics had never been heard of, the principle above adverted to and for which it was quoted, could not be denied,—not to mention that other cases are reported in which no odour has been perceptible; but then the grand object of a lachrymose declamation would have been entirely lost!*

One of the most important facts connected with the history of this poison relates to the time at which the symptoms commence,—or the period within which it begins to operate. It has been commonly laid down by writers, that the symptoms, if not immediate, commence in a few seconds or at the furthest within a minute. Important consequences may flow from the opinion adopted by a medical witness on this point. The case of Freeman adverted to by the author (p. 767) furnishes a striking instance of this. Dr. Christison candidly acknowledges, that he had given his concurrence rather too unreservedly in the opinion expressed by the majority of the witnesses on this occasion, i. e. that the deceased after taking three drachms of prussic acid, could not have had power to perform certain acts

* The line of defence in this extraordinary case appears to have been as follows: The two medical witnesses who saw the deceased are young practitioners—they have never seen a case of poisoning by prussic acid—their opinions are therefore based upon what they have read in books—these opinions may be in the main correct and founded on principles well recognised by toxicologists—let us lay aside all consideration of the principles, and attack the cases upon which the witnesses rely. This may have the effect of rendering their evidence untrustworthy with the jury, who are entirely ignorant of medical matters. If, moreover, as a legal point, no witnesses are called, it is impossible that any statements made in the defence, however erroneous in a medical view, can be contradicted by the witnesses for the prosecution. Such appears to have been the policy pursued, and it would doubtless have had the intended effect, but for the clear summing up of the judge. See Remarks on this case by Dr. Skae, Northern Journal of Medicine, May 1845.

of volition. He observes, however, "I still adhere so far to my original views, as to think it improbable that if the deceased after swallowing the poison, had time to cork the phial, wrap it in paper, pull up the bed-clothes, and place the bottle at her side, the progress of the symptoms could have been so rapid, and the convulsions so slight, as to occasion no disorder in the appearance of the body and bed-clothes; and I still likewise think, that after swallowing so large a dose, it was improbable she could have performed all the successive acts of volition mentioned above, with ordinary deliberation." (p. 768.) This is an important statement: the author evidently thinks that large doses should either destroy life so rapidly as to prevent the performance of similar acts, or if there be a short survivorship, this ought to be indicated by the body being found in a convulsed attitude, and the bed-clothes disordered. There are several cases on record which are strongly opposed to these views:—they show plainly that the body of the deceased may be found lying in a composed attitude in bed, and the clothes undisturbed under the very circumstances in which such conditions are here regarded as improbable. The only explanation which, it appears to us, can be given of the strong opinions expressed in the Leicester case, is that the facts connected with poisoning by prussic acid were then but little known.

A case which most strikingly proves the fallacy of these views has been lately reported by Mr. Hicks.* A girl swallowed an ounce of Scheele's acid, in the presence of another person who had no suspicion that she was about to take poison. She was observed to throw her arms over her head, and then to fall to the ground. She died in five minutes without having been in the least convulsed from the first; and it is worthy of remark, that she had had sufficient time to thrust the bottle into the front of her dress before the entire loss of volition. Or take the following more apposite case by Mr. Leithead.† A girl swallowed an ounce of prussic acid, recorked the phial, thrust the bottle to a full arm's length between the feather bed and the mattress, got into bed, and then drew the clothes over her body: there appeared to have been no convulsions. Cases like these appear to us to prove incontestably that the strong opinions expressed in the Leicester case were wrong; and they show that if inferences from experiments on animals are allowed to be thus applied to the human subject, they may lead to the most serious errors. Let us put the case that the prisoner had been convicted and executed upon this medical evidence; for *this* was the strongest part of the case against him, we could now only have regarded his execution as a judicial murder. The question is not whether such successive acts are performed "with ordinary deliberation," but whether they can be performed at all by a person so situated; and the facts quoted show that this is quite compatible with a much larger dose of the poison, than was taken by the female in the Leicester case. We have no doubt that in another edition, our author will see fit to give up the opinions here expressed, and no longer lend his authority to the diffusion of what we think must now be regarded as erroneous doctrines. We must do him the justice to say that in the same paragraph he qualifies his opinion, by allowing that too great importance should not be attached to his argument on the subject.

Dr. Christison states that when the quantity of poison is small, a much

* Medical Gazette, vol. i, new series, p. 462.

† Lancet, June 7, 1845, p. 640.

longer interval may elapse before the commencement of its action. (p. 769.) In proof of this, he quotes a singular case reported in the 'Edinburgh Medical and Surgical Journal' by Mr. Garson of Stromness. A gentleman took, as he supposed, about a teaspoonful of prussic acid, of which the strength was unknown; and a *quarter of an hour* elapsed, before the symptoms came on; he walked about his room and employed himself in writing in the interval. In this case, the dose, as Dr. Christison remarks, was "barely short of what is required to occasion death:" but a long protraction of symptoms may be observed, where the dose is large, a fact strikingly evinced in a case reported by Mr. Godfrey, in the 'Provincial Medical and Surgical Journal.'* A gentleman swallowed half an ounce of prussic acid, placed the bottle in the grate, walked to the top of a flight of stairs (ten paces) descended the stairs seventeen in number, and proceeded to a druggist's shop (forty-five paces), making a total of fifty-five paces and seventeen stairs. He entered the shop in his usual manner, which was slow and easy, and said in his usual tone of voice "I want some more of that prussic acid"—his eyes then became fixed with a stare, he fell and (died probably) within ten or twelve minutes from the time of taking the poison. In this case, there were no convulsions; and there was an exercise of the powers of volition and locomotion with more than ordinary deliberation. The most remarkable feature of the case was that with a decidedly fatal dose, so long an interval should have elapsed before the symptoms commenced.†

So far then with regard to the period within which the poison begins to operate. We quote these cases, because they show from the attention now paid to the subject of toxicology, that even since the publication of the present edition, important facts have come to light, which may lead materially to modify the views of the author. We shall only add that individuals may labour for a long time under the effects of this poison, and yet ultimately recover. In Mr. Garson's case (supra) several hours elapsed before recovery began to take place; and in an interesting case reported by Mr. T. Taylor of Cricklade‡ the patient lay *four hours* in a state of insensibility before recovery commenced. In this instance nearly a grain of prussic acid had been taken.

The smallest fatal dose of prussic acid, the author justly remarks, will vary with particular circumstances, such as the strength of the individual and the fulness or emptiness of the stomach at the time. (p. 770.) The Parisian epileptics were killed by a draught containing two thirds of a grain; and a patient of Dr. Geoghegan's recovered after taking the same quantity, although his life was in extreme danger. Dr. Banks of Louth met with a case of recovery in which the dose was nearly a whole grain. On referring to the report of this case, we do not find the strength of the acid stated—the patient having taken thirty drops of some kind of prussic acid. The statement of the dose in the cases of the Parisian epileptics, has been objected to, as erroneous; but on looking into the matter, it appears to us that the error, if any, arose from the very careless manner in which the case was originally reported by Orfila. A writer in the Pharmaceutical Journal, May 1845, p. 515, shows that Orfila has given

* Sept. 25, 1844, p. 398.

† See also another case of great interest by Mr. Nunneley of Leeds. Provincial Medical Journal, July 23, 1845.

‡ Medical Gazette, vol. i, new series, p. 130.

two different versions of this dose. Devergie gives a third, and Guibourt a fourth; and the only fair conclusion to be drawn from the whole statement, is that the dose actually taken is known with so little certainty, that in a medico-legal view, it can never again be made available as evidence. Dr. Christison's facts in relation to this question, require some modification, especially since certain well-observed cases bearing upon it have occurred since the publication of this edition of his treatise. The smallest dose yet known to have proved fatal to an adult, was in Mr. Hick's case*— it amounted to nine tenths of a grain; but in another instance, the same dose was taken without destroying life. It is probable that even less would suffice to kill, and although the opinion of two thirds of a grain being the smallest fatal dose, does not happen to be supported by the case quoted by our author, it is not at all improbable, that this is very near the truth.

In the treatment of poisoning by hydrocyanic acid, the author very properly advises ammonia as a diffusible stimulant and cold affusion. He refers to the antidote lately recommended by the Messrs. Smith of Edinburgh, but remarks that these cases of poisoning are commonly too rapid, to allow of the use of any chemical antidotes. In general the mouth and fauces are spasmodically closed, or the power of deglutition is entirely lost, and too much time would be consumed in the introduction of any antidote by the stomach-pump.

For the detection of the poison, distillation of the fluid is in the author's opinion the best mode of procedure. The objection that hydrocyanic acid may be formed during distillation by the decomposition of animal matter he considers "to rest upon conjecture or presumption at farthest, and I doubt whether, supposing the distillation to go on slowly in the vapour-bath, the heat is sufficient to bring about the requisite decomposition. The force of the objection must be decided by future researches." He then says "that hydrocyanic acid is apt to be formed in the course of the changes produced by various agents in organic matters. These are probably more numerous than the toxicologist is at present aware of." (p. 756.) We cannot help thinking that the author is here giving too strong a support to a doctrine which may tend to overthrow all chemical evidence in cases of poisoning by prussic acid. The admission here made of the spontaneous production of prussic acid in organic matters, will of course be applied by a skilful barrister in defence, to its formation from the contents of the stomach in the dead, or from food swallowed during life in every instance of poisoning by prussic acid. In Tawell's case Mr. Kelly thus constructed a grain of anhydrous prussic acid, found in the stomach, by supposing that the pips of apples (of which none were discovered, although sought for) furnished a fourth—"that there was some little in the cake which she (deceased) had eaten,—suppose there was some in the saliva which she must have swallowed in large quantities, when masticating the apples, and which was known to contain much prussic acid (!)—suppose (and here we think he must have rested upon Dr. Christison's statement in the above quotation,) there was some in the animal substances, and although it was stated that they would not yield prussic acid without being subjected to a greater heat than they had been subjected to, *yet it was known that when undergoing decomposition, prussic acid was constantly being evolved*," &c.! This extraordinary statement was allowed to go

* Medical Gazette, vol. xxxv, p. 893.

to the jury without any contradiction or remark except that which it received in the charge of the learned judge; and we think therefore it becomes a serious matter for inquiry, whether there be any ground for the allegation that prussic acid is a frequent or even a common result of the spontaneous decomposition which takes place in organic matters.

There is probably no man who has had greater experience on such a subject than the author of this work. We infer that he has never met with an instance, during his numerous researches for poison in organic matters, of this spontaneous formation of prussic acid, or he certainly would have recorded it, and we can only express our surprise that he should have given the slightest encouragement to a theory susceptible of such abuse without producing a solitary fact in its favour. In such a case it appears to us that we ought rather to be guided by well-ascertained facts than by chemical theories.

A process has been recently recommended by Mr. Taylor for detecting prussic acid without distillation.* If this prove successful, it will do away with at least one of the objections urged by Dr. Schubarth, namely, that hydrocyanic acid may be formed during distillation by the decomposition of organic matters, but neither this nor any other chemical process will suffice to remove the theoretical objection of the production of the poison in organic matters undergoing decomposition. The sooner therefore toxicologists direct their attention to a collection of facts in relation to this subject the better, as it involves the material issue, whether chemical evidence should or should not be received in a case of poisoning by prussic acid. If it be true, as the author asserts, that the cases of its production "are probably more numerous than the toxicologist is at present exactly aware of," we think that every counsel defending a prisoner is justified in objecting to the reception of chemical evidence, where this is based on an analysis of the contents of the stomach.

Many additions have been made to the chapter on poisonous gases, but we find nothing calling for particular notice. The same may be said of the chapters on narcotico-acrid poisoning, which have not perhaps their fair proportion compared with other parts of the work. The tests for most of these powerful alkaloids are very unsatisfactory, and this is probably the reason why the author, even in the case of strychnia and nux vomica, says but little on the subject. The work is closed with a few remarks on compound poisoning, which deserves more consideration from medical jurists than it has hitherto received.

We here close our notice of this valuable work. We have taken the liberty to differ occasionally from the author, and to point out certain facts of comparatively recent occurrence, which may render a revision of some of his opinions necessary. It is needless for us to say that the treatise furnishes the toxicologist with an ample store of well-arranged materials. It has already reached its fourth edition, and the favorable manner in which the work has been received by the profession, is a clear proof of the value attached to the labours of the distinguished author. It has now deservedly become a standard authority in courts of law, and on all difficult questions connected with toxicology, it is a work for reference to judges, barristers, and medical practitioners.

* Medical Gazette, vol. i, new series, p. 328.

